# Significance of critical closing pressures (starling resistors) in arterial circulation

**DOI:** 10.1186/s13054-024-04912-4

**Published:** 2024-04-18

**Authors:** Michael R. Pinsky, M. Ignacio Monge García, Arnaldo Dubin

**Affiliations:** 1 Department of Critical Care, University of Pittsburgh, 1215.4 Kaufmann Medical Building, 3471 Fifth Avenue, Pittsburgh, PA 15213 USA; 2 Unidad de Cuidados Intensivos, Hospital Universitario SAS Jerez, Jerez de La Frontera, Spain; 3 Facultad de Ciencias Médicas‬, Universidad Nacional de La Plata‬, La Plata, Argentina

Arterial pressure is the input pressure driving tissue blood flow. However, under most conditions organ blood flow is independent of arterial pressure. Tissue blood flow is proportional to local metabolic demand and can vary widely without any change in arterial pressure. Furthermore, changes in arterial pressure within physiologic limits do not alter tissue blood flow. The reason for these apparent incongruities derive from the determinants of organ blood flow. Tissues autoregulate their levels of delivered oxygen to meet their metabolic demand. As tissue metabolic demand increases, as occurs in the gut during digestion, the brain with cognition or muscle with exercise, local O_2_ consumption increases to sustain adequate ATP flux. This stimulates the local capillary endothelia in a retrograde fashion to decrease upstream vasomotor tone [1]. These metabolism-induced changes in local vasomotor tone are complimented by local and global sympathetic tone changes mediated through α-adrenergic receptor stimulation and systemic catecholamine release [2].

Central to the control of local blood flow is flow through vessels with tone [3]. Mean arterial pressure (MAP) remains nearly constant from aorta to peripheral arteries because most of the arterial circuit functions more as a capacitor storing blood under pressure than a conduit losing energy through flow-based resistance. Hence, radial arterial catheterization reports the same MAP as sensed in the aorta. However, as blood flows down the arterial tree to the terminal arteries and arterioles, relative flow velocity increases and vessel diameter decreases. This leads to rapid drop in arterial pressure within these small arterioles as a function of resistance. The circumferential arterial vascular smooth muscle tone opposes the expansive forces of the intralumenal vascular pressure. By Laplace’s Law vascular wall tension is a function of the ratio intralumenal pressure times the radius of curvature to wall thickness. As vessel diameter decreases for the same vasomotor tone, its active tension promoting constriction relative to intralumenal pressure increases. Once vasomotor tone exceeds local arterial pressure the vessel collapses limiting flow. The intraluminal pressure at which vessels collapse is their critical closing pressure (Pcc). Pcc is the effective back pressure to arterial flow independent of further downstream capillary and venous pressures. Starling used a pressurized external chamber through which a collapsible arterial outflow circuit flowed from his isolated heart preparations to sustain coronary perfusion; hence these collapsable vascular segments are called a “Starling resistors” [4]. Pcc is usually greater than mean systemic filling pressure (Fig. 1)[5]. The Pcc to mean circulatory filling pressure difference is called the “vascular waterfall” as flow over the edge is independent of how far it subsequently falls. Tissue perfusion pressure (TPP) is MAP minus Pcc. Accordingly, there is both arterial resistance in the small arteries that define a given arterial pressure-flow relation and a Pcc which defines the effective back pressure to that flow.

**Fig. 1:**
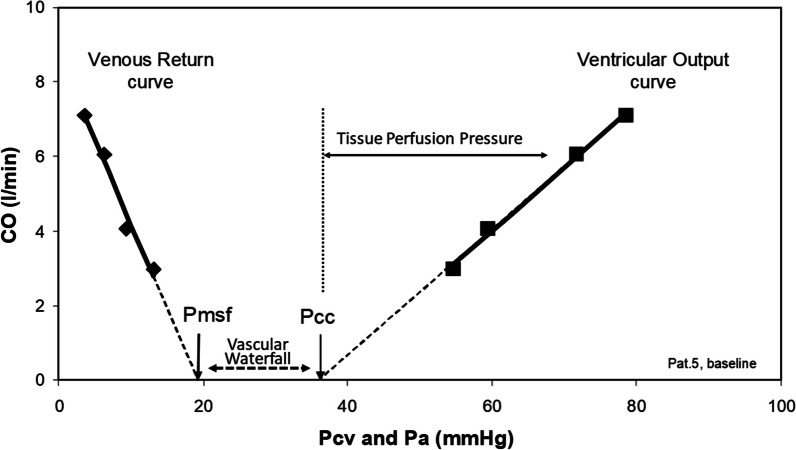
Relation between changes in cardiac output (CO) by end-inspiratory hold maneuvers and both right atrial pressure (Pra) defining the venous return curve and mean arterial pressure (Pa) defining the arterial pressure-flow relations in a ventilated anesthetized patient. Arterial critical closing pressure (Pcc) is the zero-flow extrapolation of the ventricular output curve, just as mean circulatory filling pressure (Pmsf) is the zero-flow extrapolation of the venous return curve. The pressure difference between Pcc and Pmsf is the vascular waterfall. The pressure difference between arterial pressure and Pcc is the tissue perfusion pressure. In this example from a post-operative cardiac surgery patient for a mean arterial pressure of 75 mmHg and a Pcc is 35 mmHg, the tissue perfusion pressure is (75 mmHg-35 mmHg), or 40 mmHg. (Modified from Maas et al. [5])

Arterial resistance and the collapsible segment are linked but reflect different vascular loci.. Arteriolar resistance is controlled by local and global sympathetic tone, whereas the downstream collapsible segments are influenced more by local metabolic demand. Every vascular bed has its own specific arterial resistance and Pcc. Although a lumped parameter global arterial resistance and Pcc can be defined for the arterial circuit by observing the instantaneous cardiac output to arterial pressure relation as flow is rapidly decreased, this relation may not reflect regional blood flow. Regional tissue Pcc values will vary to sustain adequate organ perfusion relative to varying local metabolic activity. If, however, there is a generalized loss of peripheral vasomotor tone, as is often seen in sepsis and post-cardiac surgery vasoplegia, global Pcc will decrease. Although this may seem like a good thing for increasing tissue perfusion, it also causes MAP to proportionally fall and any ability to autoregulate blood flow to tissues relative to their metabolic demand is profoundly blunted.

The implications of Pcc are protean to understanding cardiovascular homeostasis, regional blood flow distribution, micro-circulatory blood flow and the effect of hypotension, sepsis and vasoactive drug therapy on tissue perfusion.

First, because different vascular beds can have different resistances and Pcc, step decreases in MAP will redistribute blood flow among organs as a function of their local vascular resistances and Pcc. This explains why the kidney is vulnerable to hypotension-induced ischemia whereas the gut and the liver less so [2].

Second, both exogenous α-adrenergic agonist infusions and pathologic vasoplegia as seen in sepsis [6] will inhibit local blood flow redistribution by overriding or blocking local sympathetic feedback loops, respectively, potentially promoting tissue ischemia independent of arterial pressure.

Third, microcirculatory flow, as a direct measure of tissue blood flow, should vary independent of global changes in cardiac output and arterial pressure, as those changes are proportional changes in local metabolic demand [7]. This dissociation between measures of regional and global flow changes during sepsis and shock resuscitation may also reflect such metabolic demand imbalance as manifest by local Pcc not being responsive to local control. Increasing MAP with norepinephrine from 65 to 85 mm Hg can improve or worsen microcirculatory perfusion depending on the basal state of microcirculation [8].

In adequately fluid-resuscitated patients, evidence of tissue hypoperfusion, such as prolonged refill capillary time and oliguria with intra-abdominal hypertension, can be managed by titrating norepinephrine to higher MAP levels. Beneficial or detrimental effects of vasopressors on tissue perfusion can occur depending on their relative actions on MAP and Pcc.Monitoring TPP may offer an advantage for blood pressure optimization in circulatory shock patients [9]. In a retrospective study, lower TPP was associated with higher mortality, longer hospital stay, and higher blood lactate levels than patients with higher TPP for the same MAP [10.

Clearly, global blood flow and MAP are important hemodynamic parameters to monitor and use as initial targets for resuscitation. However, preserving arterial pressure above the population-based autoregulatory range does not insure adequate organ perfusion in the setting of vasoplegia or excess catecholamine infusions. Thus, assessment of end-organ perfusion-based function is an essential aspect in the assessment of circulatory sufficiency to define end-points of resuscitation.

## Data Availability

No datasets were generated or analysed during the current study.
